# Characterizing Inner Retinal Changes in End-Stage Inherited Retinal Diseases That Might be Suitable for Optogenetic Therapies

**DOI:** 10.1167/tvst.14.6.2

**Published:** 2025-06-02

**Authors:** Benjamin W. J. Ng, Tien-En Tan, Vasil Kostin, Robert E. MacLaren, Jasmina Cehajic-Kapetanovic

**Affiliations:** 1 Oxford Eye Hospital, Oxford University Hospitals NHS Foundation Trust, Oxford, UK; 2 Nuffield Laboratory of Ophthalmology, Nuffield Department of Clinical Neurosciences, University of Oxford, Oxford, UK

**Keywords:** optogenetics, inherited retinal disease (IRD), optical coherence tomography (OCT)

## Abstract

**Purpose:**

The purpose of this study was to characterize retinal structure in patients with late-stage inherited retinal diseases (IRD) for their suitability for optogenetic gene therapy.

**Methods:**

This was a retrospective study using clinical data and spectral-domain optical coherence tomography (SD-OCT) images of patients with late-stage IRD (visual acuity ≤ 1.0), between December 2012 and 2023 from Oxford Eye Hospital, United Kingdom. Depending on the clinical phenotype and history, the patients were divided into three groups: rod-cone dystrophy (group 1), cone-rod/cone dystrophy (group 2), and macular dystrophy (group 3). SD-OCT structural parameters including total subfoveal thickness and, if possible, individual inner layers thickness were analyzed.

**Results:**

36 patients with late-stage IRD (11, 13, and 12 in groups 1, 2, and 3) and 54 eyes (18 per group) with mean age of 55.9 ± 9.8 years and mean visual acuity of 1.72 ± 0.66 were analyzed. Mean subfoveal thickness was reduced to 167.8 ± 54.3, 153.2 ± 65.3, and 138.1 ± 41.7 µm in groups 1, 2, and 3, respectively, with no significant difference among each group (*P* = 0.33). Twenty-five of 54 eyes had well-defined inner retinal layers with mean subfoveal thickness of nerve fiber, ganglion cell, inner plexiform, and inner nuclear layers were 12.6 ± 3.9, 17.3 ± 9.9, 18.6 ± 6.7, and 29.4 ± 11.3 µm, respectively.

**Conclusions:**

In our cohort, 46.3% of degenerate retinae had preservation of the inner retina, including nerve fiber, ganglion cell, and inner plexiform layers, and/or thickening of the inner nuclear layer and may benefit from targeted cell-specific optogenetic gene therapy. Patients with indiscernible or disrupted inner layers may be amenable to a non-cell-specific approach, to target all surviving neurons.

**Translational Relevance:**

SD-OCT structural characterization of different groups of late-stage IRD offers insight into vector selection and patient eligibility for optogenetic treatments.

## Introduction

**Figure 1.**
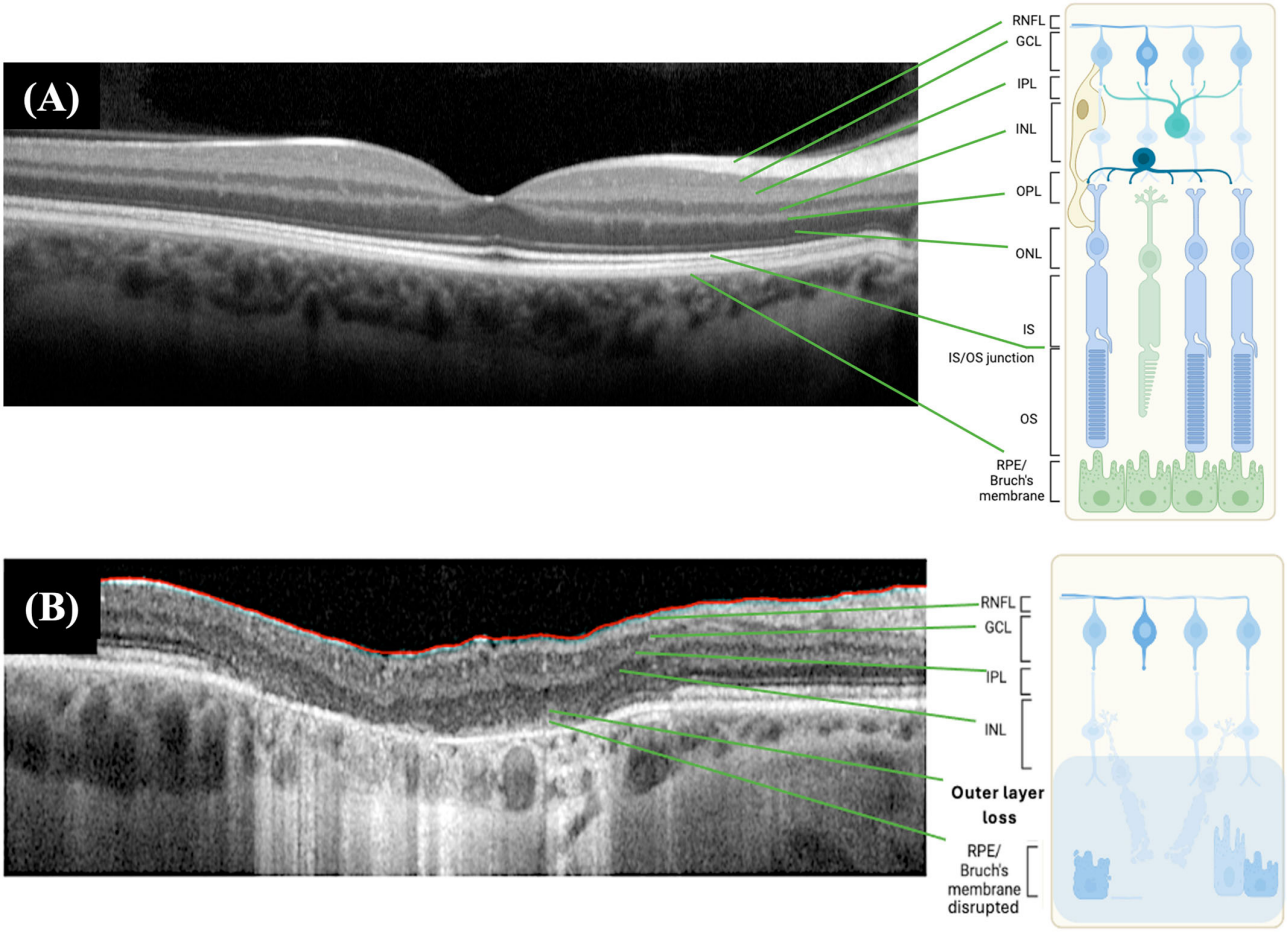
Structural differences between a healthy retina (**A**) and in a retina with advanced retinal degeneration (**B**) shown on SD-OCT. The outer retinal layer is typically lost in late stages.

Inherited retinal degenerations are a heterogeneous group of diseases that represent one of the most common causes of visual loss in children and working-age adults in many developed countries, contributing to a major disease burden to healthcare systems.^1^^–^^4^ Regardless of the genetic etiology and varying clinical progression, these disorders tend to converge to a common phenotype of outer retinal atrophy due to irreversible loss of photoreceptors but with general preservation of the inner retinal layers (Fig. 1).^5^ At this late degenerative stage, gene therapy targeting individual variants are unlikely to work and treatments are thus restricted to mutation-independent approaches via either replacing new retinal cells, for example, cell transplantation or bypassing the degenerated outer layer and stimulating the inner one through retinal implants and optogenetic therapy.^6^

In optogenetic therapy, light-sensitive proteins called opsins are ectopically expressed in the remaining cells of the inner retinal layer, allowing these cells to act as substitute photoreceptors to generate an electrophysiological signal in response to light.^7^^,^^8^ Initial results from early clinical trials using this approach have shown promising functional outcomes, with one treated patient from the PIONEER trial demonstrating improvements in visuomotor tasks on light stimulation with external goggles with correlated occipital lobe activity in the electroencephalogram (EEG) during testing.^9^ All of the current trials used an intravitreal method of delivery, with most vectors aiming to target the retinal ganglion cells (RGCs) except for the multi-characteristic opsin (MCO) trials, which aim to transduce bipolar cells through this mode (Table 1). These trials highlight the importance of preservation of the intended targets (inner layer cells) as a prerequisite selection criterion. Although there are typical structural changes in the late stages of inherited retinal diseases (IRD), loss of bipolar cells in end-stage disease and unique structural changes (e.g. deposits) in macular dystrophy must be considered.^5^^,^^10^ It is, therefore, important to characterize the aberrant inner layers of advanced degenerate retinae from a representative range of different degenerations in retinal cross-sections by spectral domain-optical coherence tomography (SD-OCT).

**Table 1.** Current Clinical Trials in Therapeutic Optogenetics for Inherited Retinal Diseases

Clinical Trial	Target Disease	Vector	Mode of Delivery	Target Cell
PIONEER Phase I/IIa open-labeled, non-randomized, dose-escalation (3 groups) trial (NCT03326336; GenSight Biologics)	Non-syndromic RP with BCVA < LP and preserved inner layers on OCT	AAV2.7m8-ChrimsonR-tdTomato Requires external light-stimulating goggles	Intravitreal	RGC
RESTORE Phase IIb, randomized, double-masked, sham-controlled trial (NCT04945772; Nanoscope Therapeutics Inc.)	Advanced RP with FrACT BCVA < 1.9 logMAR in study eye and < 1.6 logMAR in fellow eye	AAV2-MCO (multi-characteristic opsin; molecular details undisclosed)	Intravitreal	Bipolar cells
STARLIGHT Phase II, open-labeled, non-randomized single-group trial (NCT05417126; Nanoscope Therapeutics Inc.)	Advanced Stargardt disease with BCVA 1.3–1.9 LogMAR and < 20/200 in fellow eye with preserved inner layers on OCT		Intravitreal	Bipolar cells
RST-001 Phase I/IIa (NCT02556736; RetroSense Inc – acquired by AbbVie in 2020)	Advanced RP with BCVA < HM in study eye, 20/200 – CF in fellow eye	AAV2-Channel-rhodopsin 2	Intravitreal	RGC
BS01 Phase I/II safety and efficacy trial (NCT04278131; Bionic Sight LLC)	Confirmed RP with light perception in at least one eye	AAV2-ChronosFP	Intravitreal	RGC

AAV, adeno-associated virus; BCVA, best corrected visual acuity; CF, counting fingers; FrACT, Freiburg visual acuity test; HM, hand movements; LP, light perception; RGC, retinal ganglion cells; RP, retinitis pigmentosa.

In this study, the first aim of this paper is to understand the proportion of patients with IRD in our cohort which are amenable to potential optogenetic therapy. Whereas this is a single-centered study, our hospital is a tertiary referral unit that receives patients from all locations in the United Kingdom, and this is representative of the national population. The second aim is to characterize the SD-OCT scans from a subgroup of these patients, with different causative genes, who meet our set criteria for late-stage disease functionally and structurally. This will allow identification and quantification of the structural changes of the inner retinal layers in different clinical phenotypes of IRD (rod-cone dystrophy, cone-rod/cone dystrophy, and macular dystrophy). As there may be distinct changes in the inner layers of different IRD groups, this information will guide the development of suitable optogenetic treatment targeting different structural phenotypes, as well as allow clinicians to decide on patient suitability for such upcoming novel treatments, which includes the choice of target cells to photosensitize.

## Materials and Methods

### Study Design

This is a cross-sectional study of patients attending the retinal genetics clinic at the Oxford Eye Hospital (United Kingdom) from December 2012 to December 2023. A list of patients, who were coded with “rod-cone dystrophy,” “cone-rod dystrophy,” “cone dystrophy,” “macular dystrophy,” “Stargardt disease,” and “pattern dystrophy” were initially generated via the audit suite function of Medisoft electronic patient record system (Medisoft Limited, Leeds, UK). Their charts and SD-OCT (Spectralis, Heidelberg Engineering, Heidelberg, Germany) scans were systemically screened and duplicates were removed.

From an initial result generated by this keyword audit search, patients that met the criteria (described below) were consecutively selected to fill an equal number of eyes in each category. To ensure a wider variety of genes were included, the molecular diagnosis was assessed to ensure no more than five of the same genes, in each group, were causative. If a patient was removed by this manner, this was replaced by the next consecutive patient that met the selection criteria.

The study design of this retrospective analysis adhered to the tenets of the Declaration of Helsinki.^11^ Written informed consent was obtained from all patients for genetic testing and retinal imaging.

### Classification of Disease

**Figure 2.**
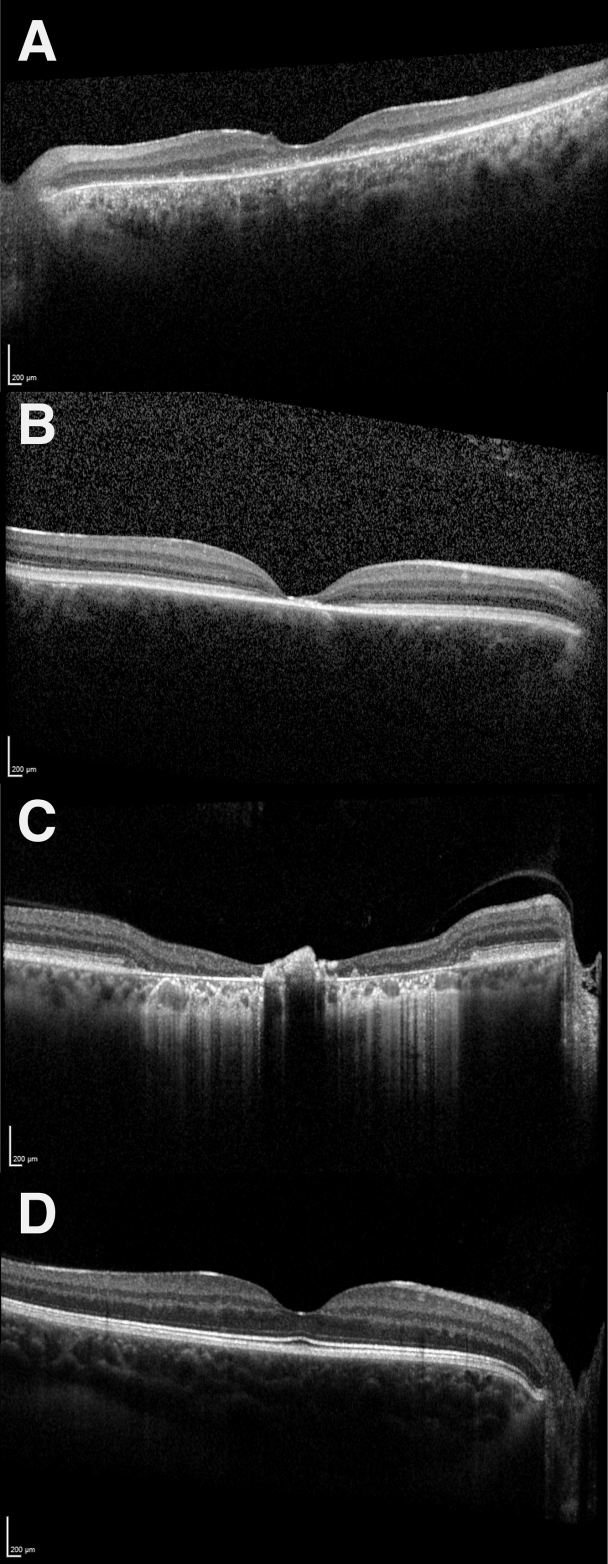
Examples of each IRD group representing (**A**) group 1: rod-cone dystrophy (*USH2A*), (**B**) group 2: cone-rod dystrophy (*KCNV2*), and (**C**) group 3: macular dystrophy (*ABCA4*). SD-OCT scan of a normal retina (**D**).

From the generated list, patients were categorized into three main IRD groups: rod-cone dystrophy (group 1), cone-rod or cone dystrophy (group 2), and macular dystrophy (group 3) based on a combination of (1) available genetic diagnosis and/or (2) clinical history and phenotype based on expert input (consultant ophthalmologist specializing in IRD) (Fig. 2, Supplementary Fig. S1).

### Inclusion Criteria

In each group, adult patients (aged at least 18 years old) with best corrected visual acuity (BCVA) ≥ 1.0 logMAR (≤20/200), which represented functionally advanced disease, were included. This was based on the inclusion criteria for clinical trials for end-stage degenerations and our BCVA inclusion criteria was less stringent to include patients that may potentially benefit from optogenetic treatment at a slightly earlier stage of the degeneration.

### Exclusion Criteria

Patients with a diagnosis of (1) acquired outer retinal diseases such as vascular causes, (2) age-related macular degeneration (AMD), and/or (3) optic neuropathies including advanced glaucoma were excluded. Patients with poor baseline vision from amblyopia were not included.

### Scan Selection Criteria

Scans of good image quality and retinal structural quality were selected. The specific parameters and examples were outlined in Supplementary Tables S1, S2 and Supplementary Figure S2.

### Investigated Parameters

For each group, the following patient demographics were (1) age, (2) gender, (3) mean BCVA (logMAR and Snellen), (4) lens status, and (5) genetic diagnosis. For ultra-low vision, the visual acuity parameter was converted into logMAR using the established formula in the literature.^12^^–^^14^

For each group, the following OCT imaging features were collected: (1) central subfield thickness, (2) parafoveal subfield thickness, (3) thickness of individual inner retinal layers (central and parafoveal, if possible), (4) thickness of the outer layer (central and parafoveal, if possible), and (5) pertinent structural features including absence of ellipsoid zone (EZ) and external limiting membrane (ELM), presence of cystoid macular edema (CME) or micro-cystoid macular edema, presence of epiretinal membrane (ERM), Bruch's membrane disruption, and foveal deposits or scarring.

### Manual Segmentation and Measurement of the Retina in SD-OCT

**Figure 3.**
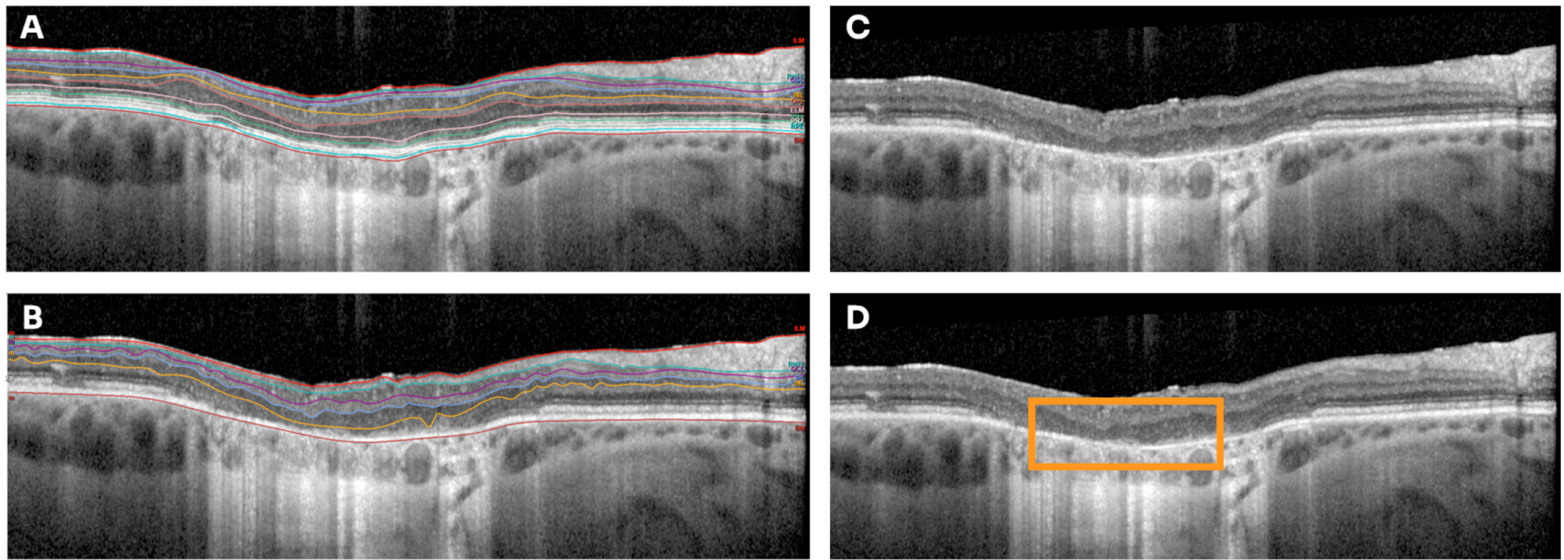
Auto-segmentation errors with HEYEX software. Incorrect OCT auto-segmentation of individual layers in a case of advanced *PRPH2* macular dystrophy (**A**). Difficulties with manual segmentation result from indiscernible layers (**B**). In many cases, mainly pertaining to the outer layers, these are impossible to accurately segment and are omitted (**C**, **D**). However, inner layers tend to be more easily distinguishable and can be manually segmented.

The Heidelberg HEYEX software automatically segments the outer (Bruch's membrane) and inner (inner limiting membrane) boundaries of the retina to determine subfield thickness, which is usually inaccurate for extremely abnormal retinae. Therefore, each SD-OCT scan was reviewed to ensure the boundary lines were placed at the correct anatomic position. Additionally, as the sub-segmentation feature was also inaccurate in such retinae, the inner retinal layers and the inner nuclear layer (INL) / outer plexiform layer (OPL) demarcation point were manually adjusted (Fig. 3). In cases where these sublayers were impossible to distinguish from one another, the segmentation was not performed, and this was noted.

Once manually segmented as accurately as possible, the foveal and parafoveal subfield thickness were determined and compared to established values in the literatures.^15^^,^^16^

The manual segmentation of retinal layers in one subgroup (cone-rod dystrophy) was performed by two independent readers (one resident and one board-certified ophthalmologist).

### Statistical Analysis

Descriptive statistics were calculated with Prism version 10.2 for MacOS (GraphPad Software, Boston, MA, USA). The Shapiro-Wilk test was used to assess the normality of SD-OCT parameters in each group. For nonparametric group data, the Mann-Whitney *U* test was used for comparison of two independent groups, and the Kruskal-Wallis test was performed if there were more than two independent groups to compare. For inter-reader agreement, intraclass (Pearson) correlation was used. These were calculated using SPSS version 29.0.1.0 (IBM, Armonk, NY, USA).

## Results

### Patient Characteristics

After an initial screen of 373 patients with IRD, a total of 36 patients and 54 eyes were selected. The demographics of our cohort of patients with late-stage IRD are summarized in Table 2. No phakic patients had cataracts of sufficient density and no pseudophakic patients had posterior capsular opacification that affected OCT imaging quality or visual acuity. One patient had *RPE65* gene supplementation, and the worse eye was included, as this treatment may not preclude future optogenetic therapy. Patients were also screened for a history of inflammatory diseases which may be a predictor of inflammation with gene therapy.^17^^,^^18^ No patients had systemic inflammatory diseases or experienced previous episodes of uveitis, including the patient who had previous gene therapy treatment.

**Table 2.** Late-Stage IRD Cohort Demographics at Last Visit

Parameters	Group 1: Rod-Cone Dystrophy	Group 2: Cone-Rod or Cone Dystrophy	Group 3: Macular Dystrophy	Overall
Patients	11	13	12	36
Eyes	18	18	18	54
Age (range)	60.2 ± 18.7	51.5 ± 17.6	58.2 ± 23.3	56.4 ± 19.8
	(21–89)	(26–80)	(22–87)	(21–87)
Gender	M: 5	M: 5	M: 6	M: 16
	F: 6	F: 8	F:6	F: 20
Mean BCVA in	2.26 ± 0.54	1.64 ± 0.59	1.15 ± 0.18	1.72 ± 0.66
logMAR/Snellen	(1.18–3.00/	(1.00–2.70/	(1.00–2.30/	(1.00–3.00/
(range)	20/302 – NLP)	20/200 – LP)	20/200 – HM)	20/200 – NLP)
Genetic diagnosis (if identified)	*RPGR* (3), *IMPG2* (2), *RP2* (1), *RP11* (1), *RPE65* (1), *USH2A* (1), *SNRNP200* (1), unsolved (1)	*CNGB3* (2), *KCNV2* (2), *PRPF3* (1), *RDH12* (1), *BBS1* (1), *ABCA4* (1), *C2orf71* (1), *PDE6C* (1), *GUCY2D* (1), unsolved (1)	*ABCA4* (5), *PRPH2* (1), *BEST1* (1), *CRX* (2), *RDH12* (1), mitochondrial (1), unsolved (1)	–
Lens status	Phakic (no significant cataracts): 7	Phakic (no significant cataracts): 16	Phakic (no significant cataracts): 16	Phakic (no significant cataracts): 39
	Pseudophakic: 11	Pseudophakic: 2	Pseudophakic: 2	Pseudophakic: 15
Previous episodes of inflammation	0	0	0	0
Previous IRD treatment	1 patient (1 eye) treated with Luxturna	–	–	1

CF, counting fingers; F, female; HM, hand motions; LP, light perception; M, male; NLP, no light perception.

### Segmentation of Retinal Layers on OCT

In this cohort, 50 of 54 (92.3%) SD-OCT scans required manual segmentation. 42 of 54 (77.7%) scans required manual correction of the Early Treatment Diabetic Retinopathy Study (ETDRS) grid to be centered at the fovea. Of these, 15 of 18 were in the rod-cone dystrophy, 15 of 18 in the cone-rod dystrophy, and 12 of 18 in the macular dystrophy groups. There was poor delineation of outer retinal layers in all scans. There was also poorly demarcated INL/OPL in 20 of 54 (37.0%) scans. Twenty-five of 54 (46.3%) eyes had well-defined inner retinal layers that were amenable to sub-segmentation (2 in group 1, 9 in group 2, and 14 in group 3).

### Central and Parafoveal Subfield Thickness on OCT

**Figure 4.**
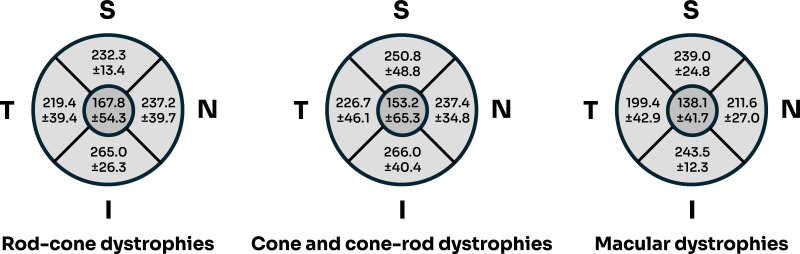
Comparison of mean CST and parafoveal thickness (with standard deviations; µm) of each IRD group after manual segmentation. Values are mapped on a modified ETDRS grid with the *inner circle* representing central 1 mm diameter. All groups showed significant thinning (normal CST ≈ 270 µm, normal parafoveal subfield thickness ≈ 332 µm). I, inferior; N, nasal; S, superior; T, temporal.

After manual segmentation of the inner and outer boundary of each retina, the mean central 1 mm subfield thickness (CST) and parafoveal subfield thickness were determined and showed atrophy in all 3 groups (Fig. 4, normal CST ≈ 270 µm and normal parafoveal thickness ≈ 332 µm).

There was no statistically significant difference between the CST in each group (*P* = 0.334). There was also no statistically significant difference between the parafoveal thickness in all four quadrants except inferiorly (superior, *P* = 0.248; nasal, *P* = 0.078; temporal, *P* = 0.326; and inferior, *P* = 0.031).

### Thickness of Individual Inner Retinal Layers

**Figure 5.**
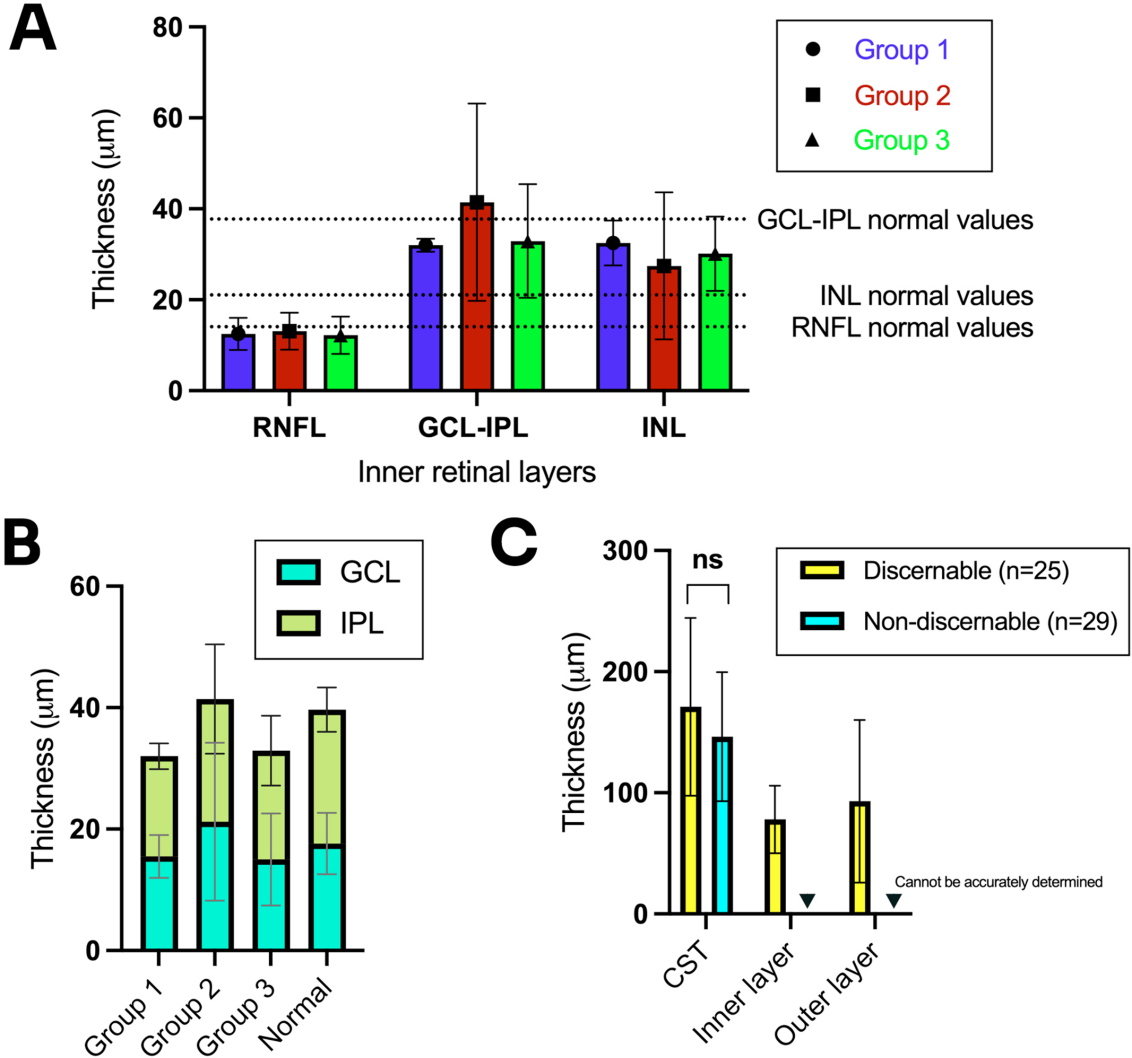
(**A**, **B**) Individual thickness of the inner retinal layers (central 1 mm) in each group. (**C**) Thickness of scans with discernible and non-discernible inner layers; ns – non-significant. *Dotted lines* represent normal age-matched values established in the literature.

The mean inner retinal layer thickness (central 1 mm) across all groups were 79.0 ± 28.0 µm (group 1 = 69.5 ± 10.6, group 2 = 82.0 ± 38.9, and group 3 = 76.2 ± 19.7 µm). After manual sub-segmentation of discernible inner retinal layers, we found that the retinal nerve fiber layer (RNFL), ganglion cell layer (GCL), and inner plexiform layer (IPL) were relatively preserved at 12.6 ± 3.9, 17.3 ± 9.9, and 18.6 ± 6.7 µm (normal = ≈14, ≈17, and ≈22), respectively (Figs. 5A, 5B). The inner nuclear layers were thickened at 29.4 ± 11.3 µm (normal = ≈21 µm; see Fig. 5A). No statistical tests could be performed to compare the thickness between groups due to the low number of eyes in group 1.

Although it was not possible to accurately determine the separate thickness of the inner and outer layers in scans with indiscernible inner layers or IPL/ONL junctions, there was no statistically significant difference between the mean CST of eyes with discernible and non-discernible inner layers (U = 299.5, *P* = 0.274; Fig. 5C).

### Inter-Reader Agreement

Due to the inaccuracy of automated segmentation in the SD-OCT software in advanced retinal degenerations, we performed manual segmentations on one group of retinal dystrophies (cone-rod dystrophy in group 2) with two independent readers. In this subset of scans used to determine inter-grader agreement, for overall retinal thickness measurements (central 1 mm), the correlation between measurements was high (*r* = 0.9856, *P* < 0.0001). For the small subset of eyes where individual retinal layer thickness measurements were possible to segment, the correlation coefficients ranged from *r* = 0.8267 to *r* = 0.9951.

### Structural Features of Degeneration on OCT

Other pertinent structural changes to the retina, which may modify the surgical procedure or affect the success of optogenetic therapy, were recorded and summarized in Table 3.

**Table 3.** OCT Structural (Non-Atrophic) Changes in Patients With Late-Stage IRD Relevant for Optogenetic Gene Therapy (Group 1: Rod-Cone Dystrophy, Group 2: Cone or Cone-Rod Dystrophy, and Group 3: Macular Dystrophy)

Features	Number (Frequency) in Each Group
Absence or	Group 1: 18/18 (100.0%)
disruption of EZ	Group 2: 18/18 (100.0%)
	Group 3: 18/18 (100.0%)
Absence or	Group 1: 18/18 (100.0%)
disruption of ELM	Group 2: 12/18 (75.0%)
	Group 3: 18/18 (100.0%)
Presence of active	Active CME:
CME or micro	Group 1: 0/18 (0.0%)
edema/cysts	Group 2: 0/18 (0.0%)
	Group 3: 1/18 (5.6%)
	Micro edema/cysts:
	Group 1: 15/18 (83.3%)
	Group 2: 14/18 (77.8%)
	Group 3: 10/17 (58.8%; 1 had CME)
Epiretinal	Group 1: 18/18 (100.0%)
membrane	Group 2: 9/18 (50.0%)
	Group 3: 12/18 (75.0%)
Disrupted Bruch's	Group 1: 1/18 (5.6%)
membrane or RPE	Group 2: 0/18 (0.00%)
migration	Group 3: 4/18 (22.2%)
Significant macular	Group 1: 0/18 (0.0%)
deposits or	Group 2: 0/18 (0.0%)
scarring disrupting inner layer structure	Group 3: 5/18 (27.8%)

CME, cystoid macular edema; ELM, external limiting membrane; EZ, ellipsoid zone.

## Discussion

Genetic therapies including gene replacement for patients with confirmed molecular diagnosis and early degeneration are developing fast and patients with *RPE65* degeneration are undergoing Luxturna gene therapy.^19^^,^^20^ Despite this, advanced degeneration for many patients with IRD and those with unconfirmed genetic mutations currently occur by early adulthood and in the working age population, representing a substantial unmet clinical and societal need. Optogenetic gene therapy represents one of the potential approaches for visual restoration in such advanced retinal degenerations, with retinal implants and cell-based therapies being the other two. Current clinical trials using this approach have only recruited patients with IRD with retinitis pigmentosa (RP) and Stargardt disease (see Table 1). Being a gene-agnostic approach, the scope of this treatment is likely to be expanded to treat almost any advanced IRD and possibly late stages of AMD.^21^ As highlighted by preclinical studies and the early trials, there are two main choices of cellular targets for optogenetic therapies: the RGC or the bipolar cells of the inner retina which would, in turn, influence the mode of delivery and vector design.^7^^,^^8^^,^^22^

The three groups of progressive IRD each have a different natural history earlier in the disease course before typically converging to central outer retinal atrophy with relative preservation of the inner retinal layer.^23^^–^^26^ As optogenetic therapy is reliant on this anatomic preservation of the inner layer, SD-OCT is the most clinically relevant and convenient imaging modality for pretreatment structural assessment. SD-OCT can also determine the integrity of the different inner retinal layers, allowing the clinician to judge if a particular optogenetic vector is suitable for that patient. Thus, in this study, we have only focused on a single modality SD-OCT analysis of patients with various causes of late-stage IRD, whom we believe may be eligible for future optogenetic therapy. Our findings confirmed the “typical” phenotype in these advanced conditions in most patients but also highlighted the structural heterogeneity that can influence optogenetic treatment. First, we confirmed there were outer layer atrophy or disruption, with EZ loss, in all patients with late-stage IRD, consistent with the natural history description in the literature (see Table 3).^27^^,^^28^ This is constant throughout all three categories of IRD, although the anatomic extent of the changes differs. Whereas both late-stage rod-cone and cone-rod/cone dystrophies have more widespread outer retinal changes, late-stage macular dystrophies tend to have such involvement mainly limited to within the central and parafoveal regions, with intact outer layers beyond the perifoveal region (see Fig. 2, Supplementary Fig. S1). This may explain the better mean BCVA in this group of patients (see Table 2). However, optogenetic therapy, like traditional gene supplementation, aims to target macula for maximum visual benefit, so this difference is unlikely to affect patient selection, and all clinical phenotypes would be potentially suitable. In line with this, one of the ongoing optogenetic trials is currently recruiting patients with macular dystrophy from advanced Stargardt disease (see Table 1).

Second, almost half of our cohort had degenerate retinae with well-defined inner retinal layers, amenable to sub-segmentation. The RNFL, GCL, and IPL were relatively preserved compared with normal age-matched values in the literature for the Spectralis OCT.^15^^,^^16^ The INL of our cohort with degenerate retinae were thickened (see Fig. 5) due to extensive photoreceptor loss, as previously described.^29^^–^^31^ We also observed RNFL preservation and, in some cases, thickening, which has been described in a few studies and is thought to be a response to the outer retinal degeneration.^30^^,^^32^^,^^33^ It was not possible to accurately distinguish the thickness of the individual sublayers of the inner retina in some eyes and up to 37.0% (20/54 eyes) had indistinguishable INL/OPL junction. These IPL, INL, and OPL sublayers are of particular interest because of the location of bipolar cells, which are possible targets. Despite the presence of indeterminate sublayers and indistinct INL/OPL junctions, there were no significant differences between the total thickness in these cases versus ones with discernible layers (see Fig. 5C). This suggests that even in most of these late-stage diseases, the typical outer retinal atrophy pattern predominates and there may still be viable inner layers to target with optogenetic therapy.

Third, we have assessed for other possible structural changes that may affect the inner retinal layer and thus delivery of optogenetic gene therapy. This includes active CME and the presence of numerous or large retina deposits or scarring. We found that, in particular, the macular dystrophy (see Fig. 2C, Table 3) may constitute a challenge to optogenetic therapy because these conditions tend to cause deposits that disrupt the inner retinal layers and eventual fibrosis of the macula as part of their pathological mechanism which often involves disruption of the basement membrane.^10^^,^^34^ As such, there is little room to target inner retinal layer cells near the fovea and a more general strategy of targeting any surviving cells for ectopic expression of an opsin may be considered instead.

The assessment of eligibility for optogenetics have previously been investigated by Jacobson et al. in a small cohort of 18 patients more than a decade ago and before optogenetics therapeutics were in clinical trial.^30^ This study only included patients with rod-cone dystrophies or Leber congenital amaurosis (LCA) harboring specific culprit genes (*DHDDS*, *CRB1*, and *CEP290*) and at least 8 eyes that with BCVA measuring better or equivalent to 6/38. This provided valuable insights into the neuroanatomic structure before irreversible degeneration, showing vision loss occurring as soon as there were ONL or EZ changes. In the eyes with advanced RP (*DHDDS* and *CRB1*) or LCA (*CRB1* and *CEP*290), the authors found preserved or thickened RNFL and GCL, consistent with their preservation in cadaveric eyes with advanced IRD.^35^^,^^36^ In contrast, our study had a wide but representative range of mutations subdivided into the three main progressive groups of IRD, and at very advanced stages mirroring the inclusion criteria of current retinal optogenetic trials. We had a less stringent criteria than these current trials, which enabled us to assess for potential candidates for treatment at a slightly earlier stage of their disease. However, despite these cohort differences, we have also found preservation and occasional thickening of RNFL and GCL. One strength of our study is the demonstration of the heterogeneity of IRD even as they converge to a typical end-stage phenotype of inner retinal preservation and outer retinal atrophy. For example, a number of macular dystrophies had more deposits or scarring which extended to the inner retinal layer and some cone-rod dystrophies had preserved ELM even at end-stage diseases (see Table 3). The earlier study by Jacobson et al. managed to illustrate this with late-stage *CRB1*-associated degenerated retinae which had prominent intraretinal hyper-reflective deposits and also classical lamination loss, the latter feature impeding sublayer thickness measurement.^30^ These gene or class-specific structural changes may affect prognosis and the type of optogenetic therapy being used in the future. Similarly, Iuliano et al. described retinal structures from OCT scans of patients with end-stage IRD in relation to assessing them for retinal prostheses.^37^ As such, their study did not focus on the measurement of inner layers, as it was not a requirement for these devices. They have found recognizable inner and outer layers in 62.5% of their cohort, 30% having indistinct inner and outer layers with identifiable RNFL, and 7.5% having no distinguishable sublayers at all. This is similar in our patient cohort where we observed well-demarcated INL/OPL in 63.0% of our scans. This finding was remarkably similar despite our two different patient cohorts, with the retinal prosthesis suitability study only analyzing patients with RP, thus suggesting a typical common late-stage phenotype.

As almost half of the degenerate retinae in our cohort were not amenable to segmentation on SD-OCT, this may not only represent a practical issue in clinics due to the need for clinicians to manually correct erroneous auto-segmentation, but also for presurgical evaluation if a specific guideline based on inner retinal layer thickness or preservation is developed. From our data, most of the poorly characterized SD-OCT scans belongs to the rod-cone dystrophy group (16/18 eyes), reflecting the larger geographic and cellular extent of these types of degenerations. The narrowing of arterioles and waxy pallor of the optic disc of typical RP may imply poorer preservation of retinal cells.^32^ On one hand, the atrophic and poorly delineated retinae in this subset of patients may predict poorer outcomes with optogenetic therapy due to the lack of viable cells for vector transduction. On the other hand, these are the patients with poorer vision and more likely in need of such treatments. We propose this group may be more suitable for optogenetic therapy that use a ubiquitous approach of targeting any surviving cells, instead of targeting a specific type of cell, and this may be achieved with a strong, nonspecific promoters in the vector. These promoters have already been used in clinical trials, as illustrated by the only currently approved ocular gene therapy, voretigene neparvovec, which uses a ubiquitous hybrid CBA promoter with a CMV enhancer, which has demonstrated long-term safety data.^19^ Alternatively, this group may benefit from targeting RGCs alone, as these are often the last cells remaining in end-stage IRD.

This study has a few limitations. First, this is a cross-sectional study and does not provide longitudinal information or suggest the optimal time for intervention. Second, the study focuses on SD-OCT with no other multi-modal imaging. However, SD-OCT is the most useful modality and only one that provides high resolution in vivo cross-sectional insight to the anatomy of the retina which other forms of imaging, such as fundus autofluorescence, do not. Adaptive optics OCT is a novel investigative tool that provides further high resolution three-dimensional structural analysis of the retina in vivo but is impractical to use in a clinical setting and especially in blind patients who cannot fixate.^38^ Second, an important consideration for the success of optogenetic is the presence of an intact post-retinal visual pathway, which we had not studied. Jacobson et al. had analyzed the optic disc OCT of patients with IRD for optogenetics suitability, showing preservation or thickening of the peripapillary nerve fibers despite poor vision. The authors have also suggested the use of functional magnetic resonance imaging (fMRI) in response to light to demonstrate residual function.^30^ However, patients with RP have previously been shown to have preservation of normal visual cortical responses on fMRI. There is also great penchant for cortical remodeling that may further stabilize the central visual circuitry.^39^^,^^40^ Furthermore, in patients with LCA2 treated with AAV2-hRPE65v2, there were intact and responsive post-retinal visual pathways on fMRI despite prolonged periods of poor vision.^41^^–^^43^ These pathways may also be tested by visual-evoked potential (VEP) or occipital EEG if there is still light perception. Even in patients with no light perception, these baseline investigations may demonstrate functional recovery with successful optogenetic treatments.^9^^,^^44^ These additional investigations were not available in our patients as it was a retrospective study and are also not routinely performed in a retinal genetics clinic. Third, using BCVA as a criterion for late-stage degenerations may introduce bias for the different groups of IRD as macular dystrophies disproportionately affect the central vision earlier than the other two groups and thus blurring the definition of “late-stage” disease. However, this parameter is the established recruitment criteria for the current optogenetic trial for Stargardt disease. In our cohort of macular dystrophy, the mean BCVA is 1.15 logMAR (20/282), which is close to the recruitment criteria (see Table 1, Table 2). Finally, we have not included other forms of retinal degenerations or choroidal dystrophies, such as AMD and choroideremia, respectively, which each present their own unique and separate challenges to optogenetic therapy.^21^^,^^45^

## Conclusions

Optogenetic therapy is a promising gene agnostic approach for visual restoration in end-stage inherited retinal degenerations. Characterization of inner retinal changes by SD-OCT offers insight into vector design and development of guidelines for patient selection for future optogenetic treatments. Advances in machine learning may lead to development of a software that can accurately and automatically segment OCT sections of degenerate retina in the future, aiding further analysis and presurgical evaluation. It would also be interesting for future works to obtain optic nerve OCT scans to determine peripapillary retinal nerve fiber layer thickness from a broad range of patients with late-stage IRD in order to provide some insights on the integrity of these afferent fibers, as well as functional tests (e.g. VEP, EEG, or fMRI) to assess an intact visual pathway.

## Supplementary Material

Supplement 1

Supplement 2
