# Supplementary material for: Characterizing Inner Retinal Changes in End-Stage Inherited Retinal Diseases That Might be Suitable for Optogenetic Therapies
Source: Transl Vis Sci Technol. 2025 Jun 2;14(6):2. doi: 10.1167/tvst.14.6.2 (PMC12136128; doi:10.1167/tvst.14.6.2)

Supplementary Figures and Tables

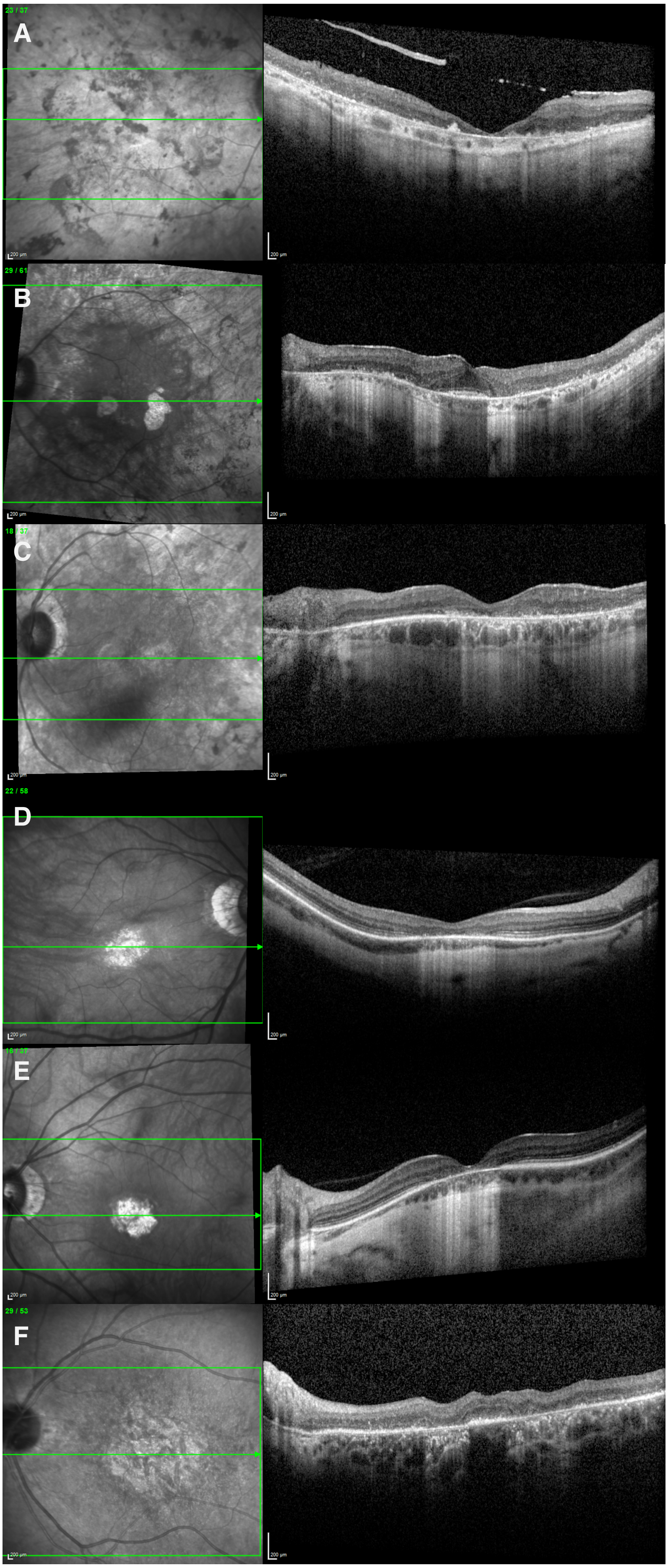

Fig S1. Further examples of high-quality centred OCT scans typical of: rod-cone dystrophy (Group 1 A-B), cone-rod dystrophy (Group 2, B-C), and macular dystrophy (Group 3, E-F). Causative mutations for each image: (A) *ARL3* X-linked rod-cone dystrophy (RP2), (B) *RPGR* X-linked rod-cone dystrophy (RP2), (C) *BBS1* cone-rod dystrophy in Bardet-Bidel syndrome, (D) *GUCY2D* variant leading to cone-rod dystrophy, (E) *ABCA4* Stargardt disease, (F) *CRX*-associated macular dystrophy.

Table S1. Inclusion criteria for segmentation in late-stage retinal degenerations.

General quality	• No significant noise<br>• No significant media opacities<br>• Good centration (not out of frame and ETDRS grid can be adjusted to fit to frame)<br>• No motion artefacts<br>• No signal loss e.g. blinking
Retinal structure quality	• High contrast between inner retinal layers – visible and distinguishable<br>• No significant disruption e.g. by deposition or scarring<br>• Minor shadowing or artefacts may be present but does not affect layer visibility

Fig S2. Examples of poor-quality scans excluded from analysis (did not meet inclusion criteria in S1).

<u>High noise</u>

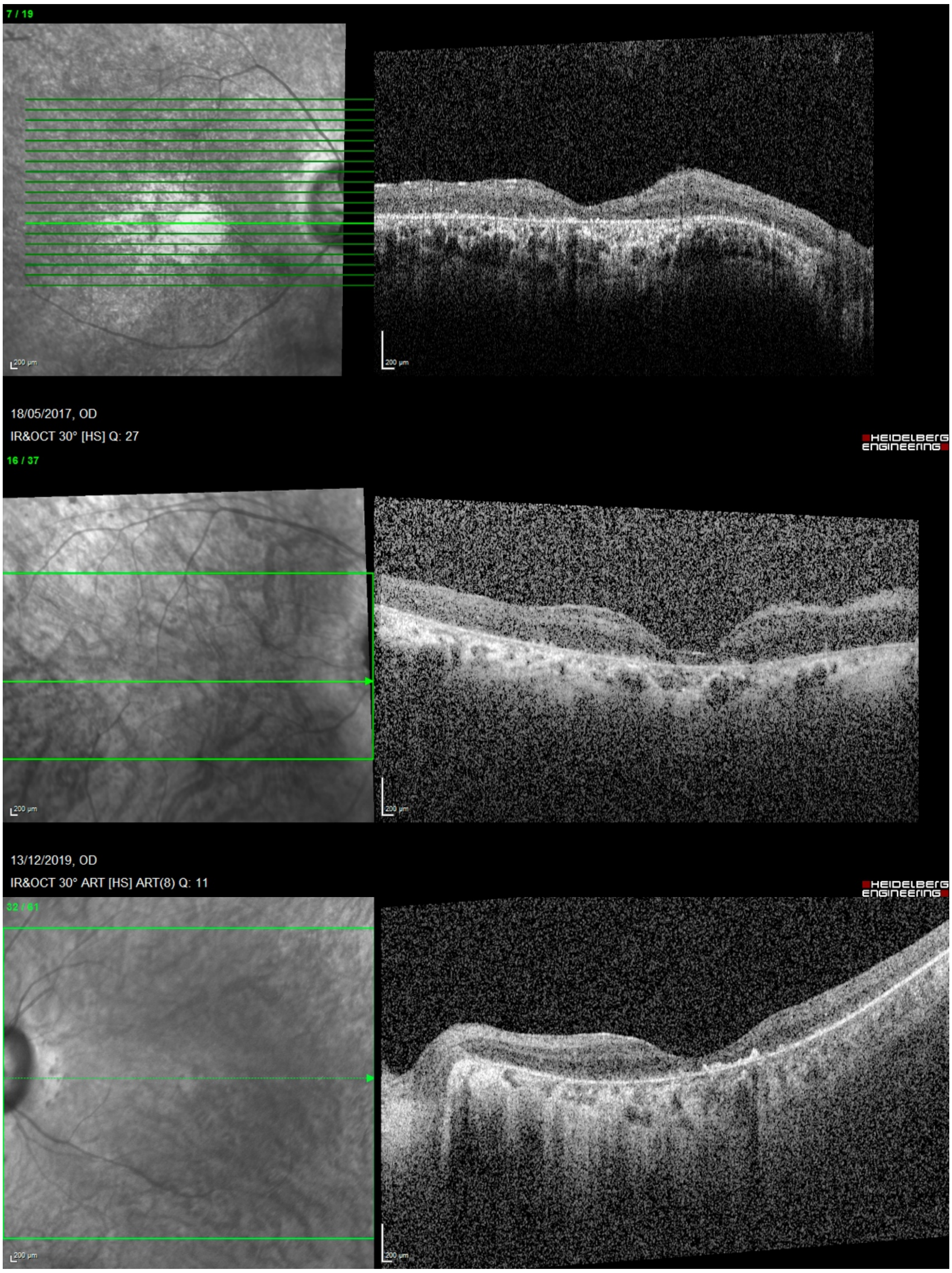

<u>**Layers indistinguishable**</u>

Scan with high myopia and scarring disrupting the inner retinal layers.

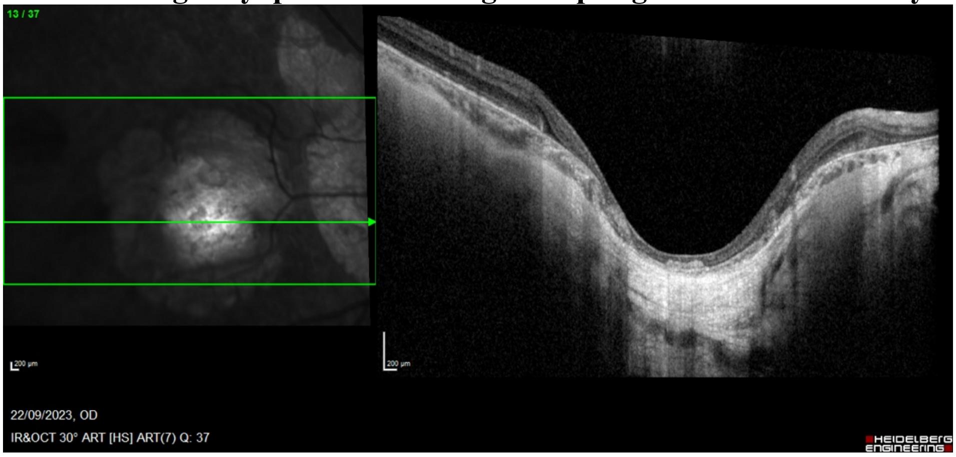

Significant disruption of the retinal structure with deposition.

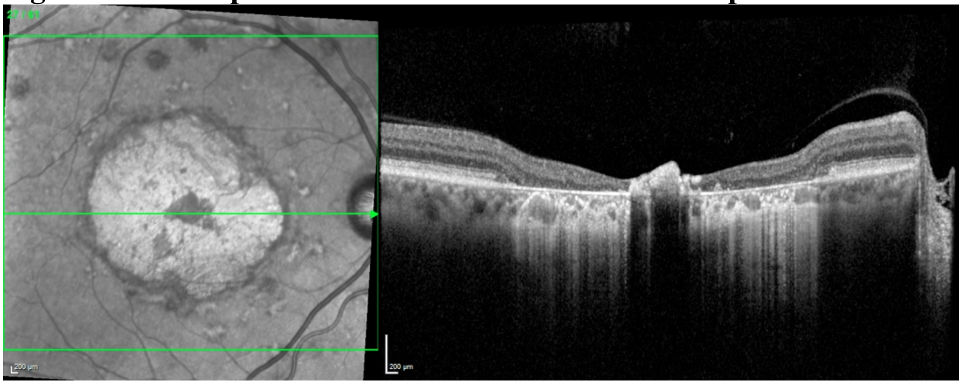

Optical gap which may affect INL segmentation but other layers may still be segmentable (one author segmented and another did not).

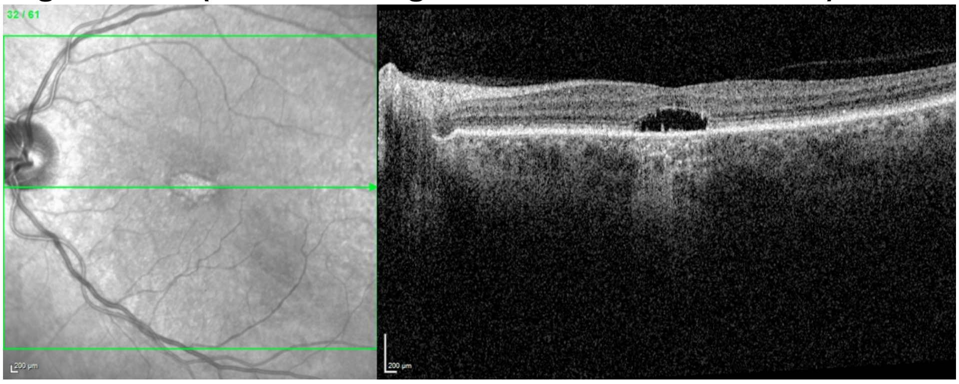

High quality scan with poor laminations, with every layer being indistinguishable.

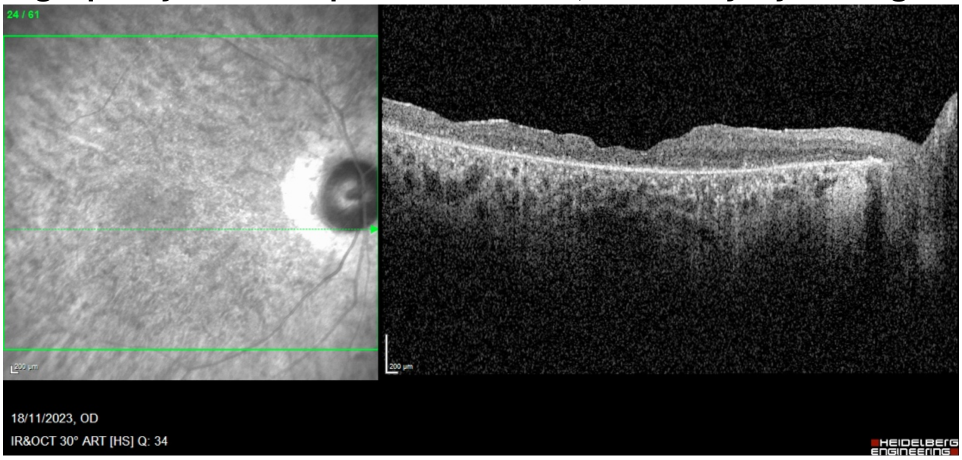

Complete atrophy: impossible to segment inner layers.

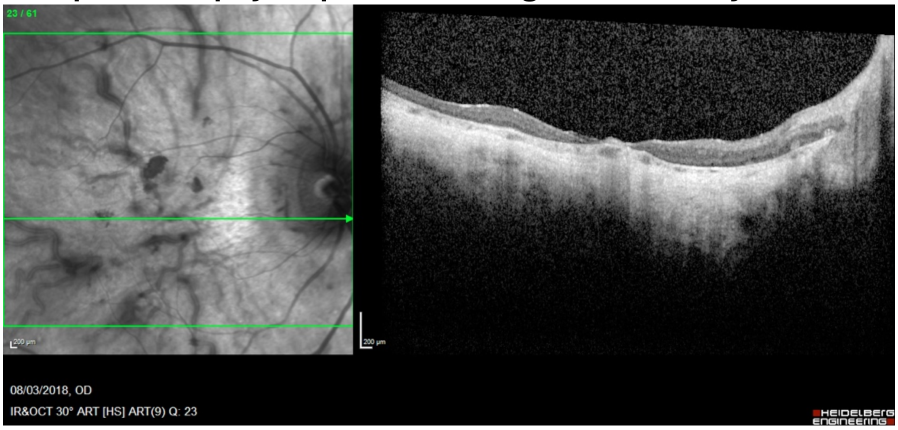

<u>Poor centration</u>

Example of poor automated centration which has to be manually corrected. Therefore, it has to be re-adjusted in order to determine the central and parafoveal retinal thickness accurately. However, because of the parafoveal fluid cyst, which disrupted layers nasal to the fovea, this scan was not deemed segmentable for the inner retinal layers. The bottom figure illustrates the Raster line at the correct position (with the original unadjusted automated ETDRS grid) where the fovea is located.

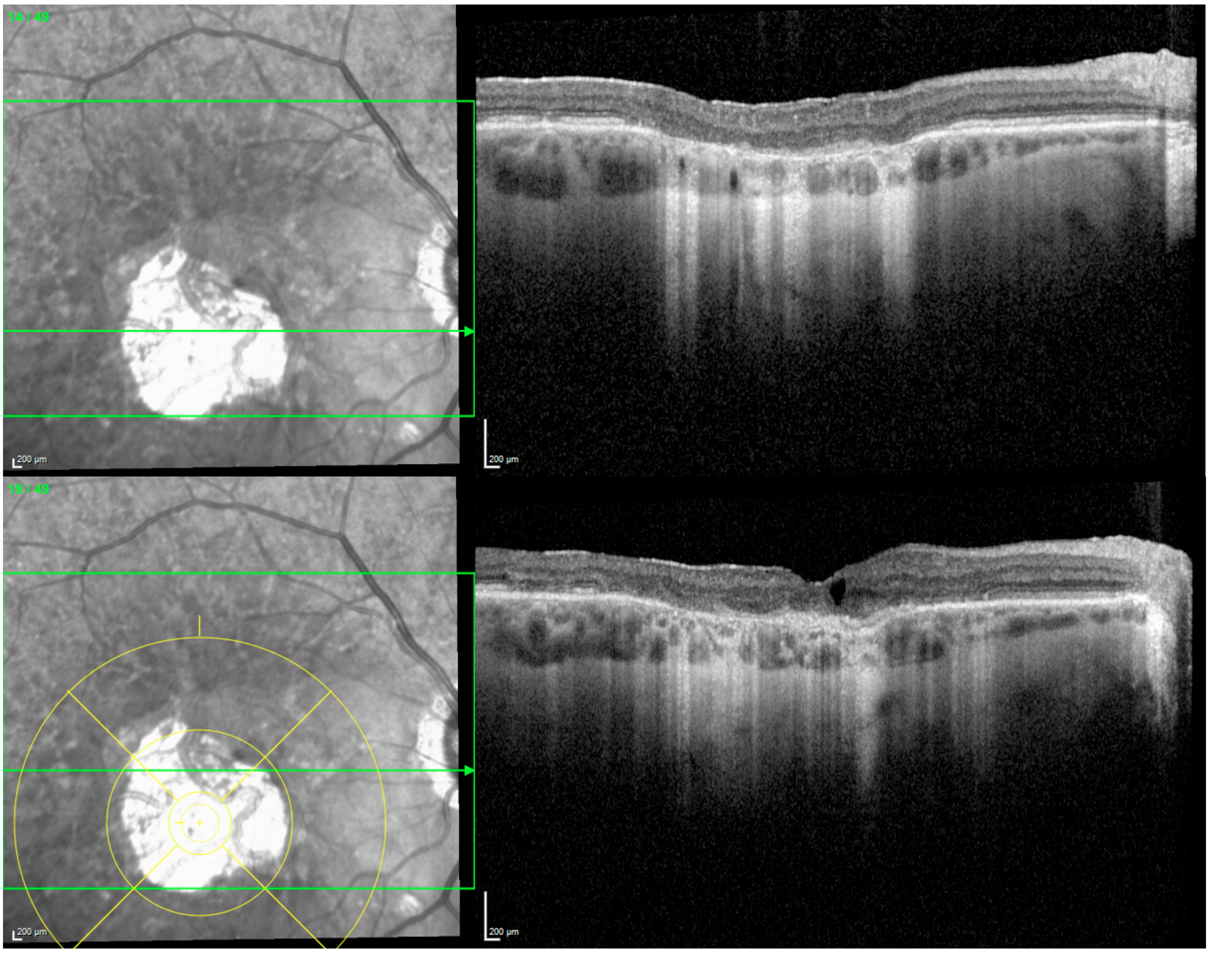

Supplement: Supplement 1 [file tvst-14-6-2_s001.pdf]
