# Supplementary material for: Characterizing Inner Retinal Changes in End-Stage Inherited Retinal Diseases That Might be Suitable for Optogenetic Therapies
Source: Transl Vis Sci Technol. 2025 Jun 2;14(6):2. doi: 10.1167/tvst.14.6.2 (PMC12136128; doi:10.1167/tvst.14.6.2)
Supplement: Supplement 2 [file tvst-14-6-2_s002.pdf]

Phenotype/ patient ID (pseudo-anonymised)	Laterality	Number of B scans	Central 1 mm B scan number	Segment-able B scans (1: yes, 0: no, ?: unclear)	Exclusion	Notes/ consensus with first author (BN)
MD6	OD		11	1		
MD6	OS	40		1		
CRD7	OD	37		0		
RCD9	OS	19		1		
CRD5	OD	37		0		
CRD5	OS	37		0		
MD8	OS	61		1		
CRD4	OD	61		0		
CRD8	OD	37		1		
CRD8	OD			1		
CRD3	OS	36		0	Retinal layers are not distinguishable in ROI due to high noise	
CRD13	OS	61		1	RNFL, GCL, IPL are possible to segment, INL challenging especially on top of the gap	
RCD7	OD	19		0	High noise, retinal layers are not distinguishable	
RCD7	OS	19		0	High noise, retinal layers are not distinguishable	
RCD6	OD	61		0	High noise, retinal layers are not distinguishable	
RCD6	OS	61		0	High noise, retinal layers are not distinguishable	
RCD2	OD	37		0		
RCD2	OS	37		0		
RCD5	OD	49		1		
RCD10	OD	37		0	Inner retinal layers not distinguishable	
RCD10	OS	37		0	Some scans inner layers are distinguishable but hard to segment.	
MD12	OD	37		1		
MD12	OS	37		1		
MD7	OD	61	11	1		**BN: not possible to segment - deposits**
RCD4	OD	121	19	1	Most of the inner retina in central 1mm is segmentable	
RCD11	OD	19	5	0	High noise, retinal layers are not distinguishable	
MD1	OD	37	9	1	High noise but segmentable	
MD1	OS	37	9	1		

CRD9	OD	61	11		1		
CRD9	OS	61	11		1		
MD2	OD	37	11		1		
MD2	OS	37	11		1		
RCD8	OD	61	9		0	Some layers are distinguishable, but very high noise	
RCD8	OS	61	9		0	Some layers are distinguishable, but very high noise	
MD5	OD	37			1	Not included in analysis	
MD5	OS	37	10		1		
MD4	OS	37	12		1		
MD9	OD	37	10		1		
MD9	OS	37	10		1		
CRD1	OD	37	10		0	inner retinal layers are almost fully degenerated	
CRD1	OS	37	11		0		
RCD3	OD	37	10		0	inner retinal layers are almost fully degenerated	
RCD3	OS	37	10		0	inner retinal layers are almost fully degenerated	
CRD10	OD	58	10		1		
MD11	OD	37	10	?			**BN: possible to segment**
CRD2	OD	37	10		1	not all scans but some of them are segmentable	
CRD2	OS	37	10	?		very high noise, a few scans segmentable but most of them not	
CRD12	OD	61	10		1		
MD10	OD	37	11	?			**BN: Not possible to segment**
MD10	OS	37	11		0	very high noise	**BN: Not possible to segment**
RCD1	OD	61	11	?		some parts are possible to segment	
RCD1	OS	61	11		1	most parts are possible to segment	
MD3	OD	49	9		1		
CRD11	OD	37	9		1		
CRD6	OS	37	9		1		

MD: macular dystrophy
CRD: cone-rod dystrophy
RCD: rod-cone dystrophy
